# Chemical Characterization of Three Accessions of *Brassica juncea* L. Extracts from Different Plant Tissues

**DOI:** 10.3390/molecules25225421

**Published:** 2020-11-19

**Authors:** Yassine Oulad El Majdoub, Filippo Alibrando, Francesco Cacciola, Katia Arena, Eleonora Pagnotta, Roberto Matteo, Giuseppe Micalizzi, Laura Dugo, Paola Dugo, Luigi Mondello

**Affiliations:** 1 Department of Chemical, Biological, Pharmaceutical and Environmental Sciences, University of Messina, 98168 Messina, Italy; youladelmajdoub@unime.it (Y.O.E.M.); arenak@unime.it (K.A.); pdugo@unime.it (P.D.); lmondello@unime.it (L.M.); 2 Chromaleont s.r.l., c/o Department of Chemical, Biological, Pharmaceutical and Environmental Sciences, University of Messina, 98168 Messina, Italy; filippo.alibrando@chromaleont.it (F.A.); giuseppe.micalizzi@chromaleont.it (G.M.); 3 Department of Biomedical, Dental, Morphological and Functional Imaging Sciences, University of Messina, 98125 Messina, Italy; 4 CREA-Council for Agricultural Research and Economics, Research Centre for Cereal and Industrial Crops, 40129 Bologna, Italy; eleonora.pagnotta@crea.gov.it (E.P.); roberto.matteo@crea.gov.it (R.M.); 5 Department of Sciences and Technologies for Human and Environment, University Campus Bio-Medico of Rome, 00128 Rome, Italy; l.dugo@unicampus.it; 6 BeSep s.r.l., c/o Department of Chemical, Biological, Pharmaceutical and Environmental Sciences, University of Messina, 98168 Messina, Italy

**Keywords:** *Brassica juncea* spp., metabolites, foods, volatile, non-volatile, nutraceuticals, HPLC, GC

## Abstract

Indian mustard or *Brassica juncea* (*B. juncea*) is an oilseed plant used in many types of food (as mustard or IV range salad). It also has non-food uses (e.g., as green manure), and is a good model for phytoremediation of metals and pesticides. In recent years, it gained special attention due to its biological compounds and potential beneficial effects on human health. In this study, different tissues, namely leaves, stems, roots, and flowers of three accessions of *B. juncea*: ISCI 99 (Sample A), ISCI Top (Sample B), and “Broad-leaf” (Sample C) were analyzed by HPLC-PDA/ESI-MS/MS. Most polyphenols identified were bound to sugars and phenolic acids. Among the three cultivars, Sample A flowers turned were the richest ones, and the most abundant bioactive identified was represented by Isorhamnetin 3,7-diglucoside (683.62 µg/100 mg dry weight (DW) in Sample A, 433.65 µg/100 mg DW in Sample B, and 644.43 µg/100 mg DW in Sample C). In addition, the most complex samples, viz. leaves were analyzed by GC-FID/MS. The major volatile constituents of *B. juncea* L. leaves extract in the three cultivars were benzenepropanenitrile (34.94% in Sample B, 8.16% in Sample A, 6.24% in Sample C), followed by benzofuranone (8.54% in Sample A, 6.32% in Sample C, 3.64% in Sample B), and phytone (3.77% in Sample B, 2.85% in Sample A, 1.01% in Sample C). The overall evaluation of different tissues from three *B. juncea* accessions, through chemical analysis of the volatile and non-volatile compounds, can be advantageously taken into consideration for future use as dietary supplements and nutraceuticals in food matrices.

## 1. Introduction

Brassicaceae vegetables, consumed worldwide, represent an important part of the human diet, due to their remarkable supply of health-promoting substances that can potentially reduce the risk of diseases. These vegetables are potential sources of glucosinolates, carotenoids, amino acids, vitamins (C and E), and polyphenolic compounds [1,2,3,4,5,6,7,8,9,10,11,12,13]. The most consumed vegetable species is *B. oleracea* L., which include vegetable and forage forms, such as kale, cabbage, broccoli, Brussels sprouts, cauliflower, and others. *Brassica juncea* (L.) Czern. (*B. juncea)*, also known as Indian mustard or brown mustard, is well-known for its green vegetable. It is also used as a root and leaf vegetable in China and as a condiment in Europe and America.

In general, it has been reported that a high intake of *Brassicaceae* is associated with the prevention of several cancers, such as colon and lung cancers [14]. Specifically, *B. juncea* leaves extract has shown in vitro anticancer-activity against colon and lung cancers [15], antioxidant activities [16], decrease of lipid peroxidation under diabetic oxidative stress [17], and inhibition of body fat accumulation [18]. In addition, *B. juncea* polyphenols have also shown beneficial effects concerning treatment for the cognitive disorder associated with diabetes [19].

Among bioactive molecules, flavonoids and phenolic acids are the most characterized groups in *Brassica* species. Flavonoids contain a basic structure of two aromatic benzene rings separated by an oxygenated heterocyclic ring with variation in the number and distribution of phenolic hydroxyl groups across the molecules and differences in substitution [20]. Flavonoids protect plants against UV radiation, pathogenic microorganisms, insects, and plant-feeding animals.

The most ubiquitous subclass of flavonoids in plant foods and *Brassica* crops, in general, is flavonols, with the main aglycones, quercetin, kaempferol, and isorhamnetin, are most commonly found as *O*-glycosides, whereas myricetin is less common. The flavonols occur in plant tissues as complex conjugates, with one to five sugar moieties bound to the aglycone, and they are often acylated with hydroxycinnamic acids. Variation in the polyphenolic content is related to the biosynthesis in the plant, which is influenced by many factors, such as cultivar, climate, postharvest treatments, and agricultural and environmental factors [1]. 

The nutritional interest of *Brassica* crops is partly related to their polyphenolic compound contents, which can be quite different among species and even among crops from the same species. The polyphenol composition of different *Brassica* species has been described, revealing distinct qualitative and quantitative profiles [21]. For example, total polyphenol content in edible parts of *B. oleracea* L. has been reported to be 2-fold higher than in Brussels sprouts, cauliflower, and broccoli [22]. In other papers, the content of flavonol aglycones in *B. oleracea* has been reported [23,24,25]. 

Moreover, it has already been reported that volatile compounds occurring in plant foods, besides being responsible for organoleptic properties, do present favorable properties for human health [26]. Concerning *B. juncea*, some of its volatile compounds, e.g., alkanes, ketones, and isothiocyanates, have been already described [27,28,29].

The objective of this study was to carry out the determination of the metabolite content by HPLC-PDA/ESI-MS/MS of different portions (leaves, stems, roots, flowers and seeds) of three accessions of *B. juncea,* ISCI 99 (sample A), ISCI Top (sample B), and ISCI “Broad-leaf” (sample C), in order to be implemented in the food matrices as nutraceuticals. The volatile content of the most complex samples, *viz.* leaves, was also analyzed by GC-FID/MS, along with the chemical characterization of defatted seed meals (DSM). 

Sample A, one of the first selections at CREA-CI (Bologna, Italy) [30,31], was selected for its so-called biofumigation technique, especially applied in greenhouse contexts. For this reason, its tissues present a high content of glucosinolates. Furthermore, this plant has a brief cycle and an early flowering time during summer, with a good plasticity and adaption to different pedoclimatic conditions. As the other Brassicaceae, with small seeds, Sample A can be sown during both fall and springtime, and prefers refined ground even though it can afford sod seeding. Sample B was recently registered at the Plant Variety Protection Office (PVPO) through a USDA PVP certificate (https://apps.ams.usda.gov/CMS/CropSearch.aspx). As for the older Sample A, this variety was bred and selected at CREA-CI (Bologna, Italy) for biofumigation purposes, and applied as green manure through soil incorporation. Sample C line has some interesting peculiarities with respect to the other varieties, such as a high biomass production, with a very characteristic broadleaf, even though it is more sensitive to low temperatures, pests, and diseases.

So far, only scattered information is currently present in literature concerning both volatile and non-volatile (metabolic) compositions of the *B. juncea* species. Recently, the determination of the metabolite content of the *B. juncea* accessions presented in this study was reported by the authors using comprehensive two-dimensional liquid chromatography coupled with a photodiode array and mass spectrometry detection [32].

## 2. Results and Discussion

### 2.1. Identification and Semi-Quantification of the Metabolite Content in B. juncea Cultivars by HPLC-PDA/ESI-MS/MS

**Figure 1:**
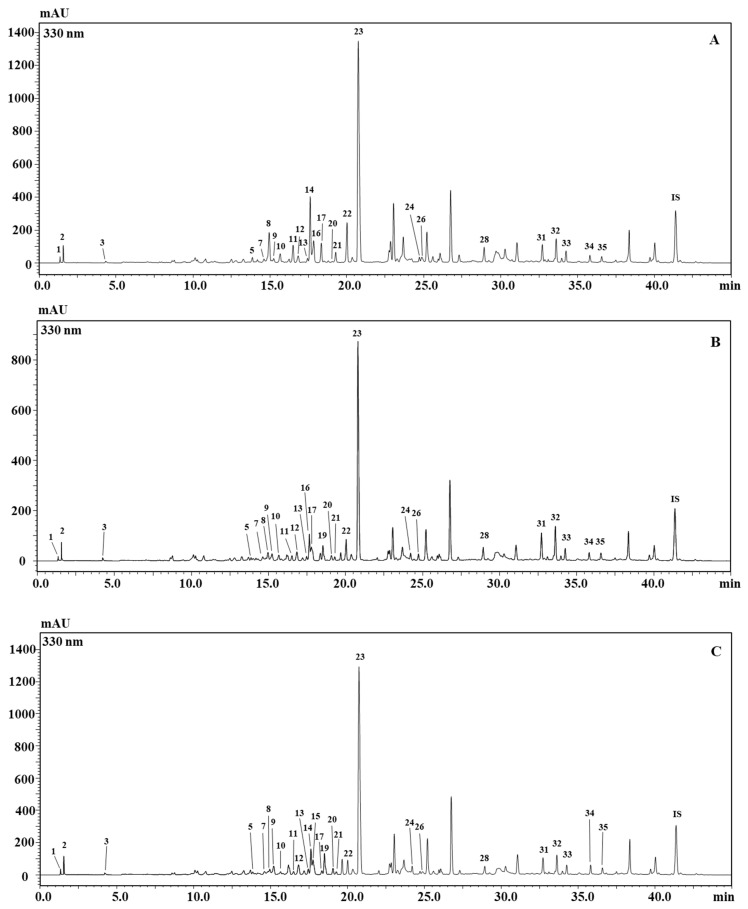
HPLC-PDA chromatograms of the metabolite content of flowers of the three *Brassica* accessions investigated. (**A**) *B. juncea* ISCI 99; (**B**) *B. juncea* ISCI Top; (**C**) *B. juncea* ISCI “Broad-leaf”.

So far, Brassicaceae metabolite composition has been widely examined [2,3,4,5,6,7,8,25,32]. The main flavonols in Brassica vegetables are reported to be *O*-glycosides of quercetin, kaempferol, and isorhamnetin. The sugar moiety found in Brassica vegetables is glucose, occurring as mono-, di-, tri-, tetra-, and pentaglucosides, also commonly found acylated by different hydroxycinnamic acids. The latter are phenolic acids occurring in Brassica vegetables, with the most common ones represented by p-coumaric, caffeic, sinapic, and ferulic acids. Figure 1 and Table 1 report the metabolite characterization of the flower extracts of the three *B. juncea* accessions described in Materials and Methods, which turned out to be the most complex ones among the samples investigated. 

Compound identification was carried out, based on retention time, UV, ESI-MS/MS spectra, and literature information. For example, Km 3-diglucoside-7-glucoside (peak 3) and Km glucoside (peak 30) had characteristic UV λ_max_ around 265 and 345 nm, whereas Qn-3-diglucoside (Peak 14) or Is-3,7-diglucoside/Is-glucoside (peak 23/peak 29) had UV absorption maxima around 256 (or plus a shoulder around 266) and 354 nm. The attachment of a hydroxycinnamoyl group to the glycosyl function leads to a shift of UV absorption maxima to 326–340 nm, while the molecular ion was increased by 162, 176, 192, and 206 Da (or the sum of two acyl groups when they occur in the glucoside) for caffeoyl, feruloyl, hydroferuloyl, and sinapoyl groups, respectively [6].

Among the identified compounds, sinapic acid and ferulic acid derivatives were the major phenolic acids, both occurring in 13 of them. Concerning flavonoids, kaempferol derivatives were the most representative ones (11), followed by quercetin (5) and isorhamnetin (2). In exception for the two isorhamnetin glucosides and quercetin 3-diglucoside, all of the other flavonoids occurred as acylated by different hydroxycinnamic acids. The sugar moiety was represented by glucose or sophorose in the form of mono-, di-, tri-, and tetra-glucosides [2,3,4,5,6,7,8,25,32]. Furthermore, the early eluting compounds were represented by malic acid and citric acid as earlier reported [32]. Compounds not detected in any of the plant tissues analyzed are labeled as Nd: not detected. 

As far as quantification is concerned, normally, the determination of Brassica spp. content is carried out after acidic and/or alkaline hydrolysis, due to the lack of commercial standards, [2,4,5]. Following the approach employed in our previous work, limited to only three samples of the different cultivars [31], semi-quantification of the native flavonoid composition of all thirty-six samples analyzed, the three cultivars of *B. juncea* was carried out by the RP-HPLC system coupled to PDA detection. Notably, due to the unavailability of corresponding reference materials, three selected standards, representatives of the distinct chemical classes, namely, Km 3-*O*-glucoside, Is 3-*O*-glucoside, and Qn 3-*O*-glucoside, were adopted, and corresponding calibration curves were prepared. Table 2 reports calibration curves, correlation coefficients (R^2^), limits of detection (LoDs), limits of quantification (LoQs), and relative standard deviations (RSDs) of the peak areas for each standard selected. R^2^ values ranged from 0.9939 to 0.9963, LoQ and LoD values ranged from 13 to 48 ppb and from 43 to 159 ppb, respectively, whereas RSD values were lower than 0.41%. 

**Figure 2:**
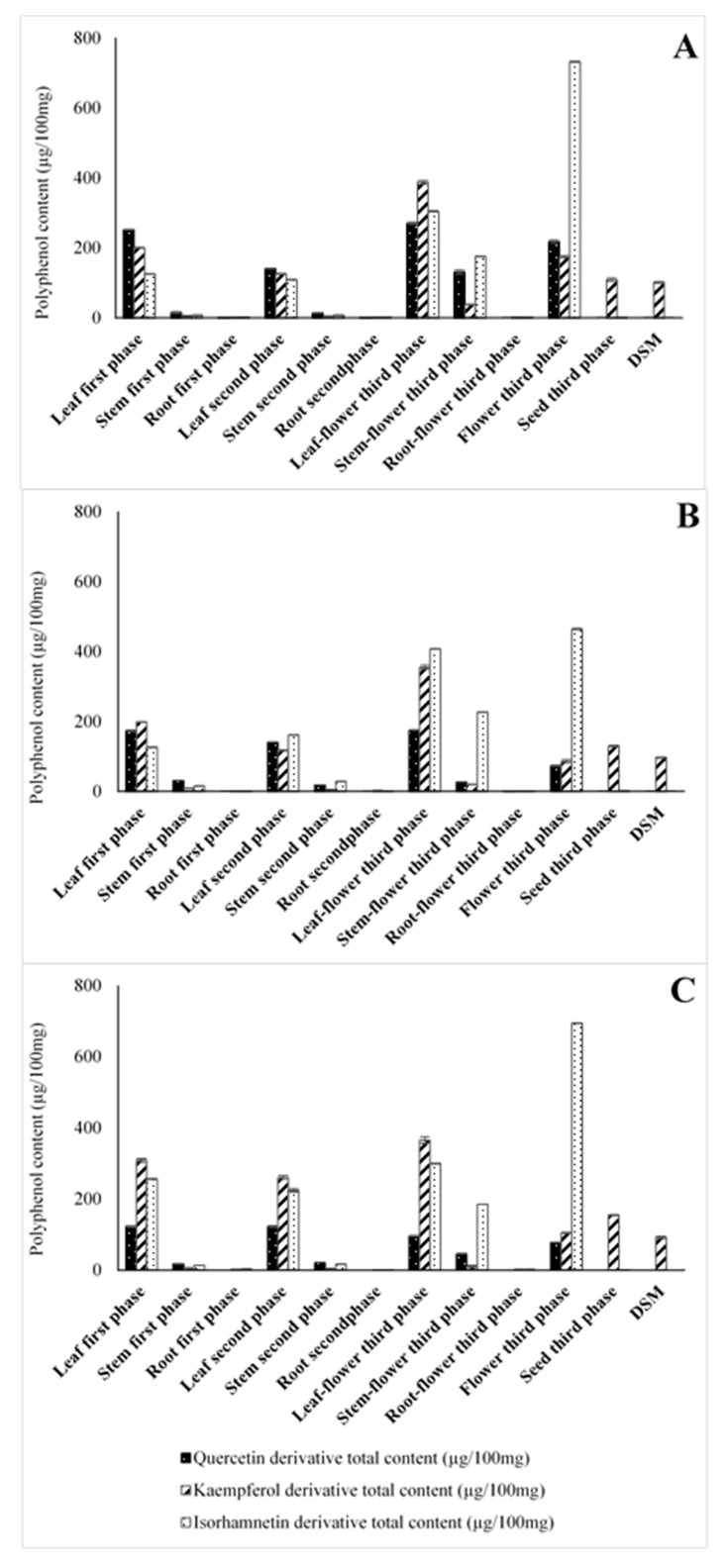
Polyphenol total content in (µg/100 mg DW) of different portions of three accessions of *B. juncea*. (**A**) *B. juncea* ISCI 99; (**B**) *B. juncea* ISCI Top; (**C**) *B. juncea* ISCI “Broad-leaf”.

Flavonoid determination in the three *B. juncea* breeding lines is reported in Figure 2, and Table 1 and Tables S1–S3. Among all samples tested, the flowers presented the highest flavonoid content (Sample A, 1124.69 µg/100 mg dry weight (DW); Sample B, 623.78 µg/100 mg DW; Sample C, 876.45 µg/100 mg DW). Considering the three different flavonoid classes, Is derivatives were the most abundant in all the three cultivars: Sample A (732.24 µg/100 mg), Sample C (694.30 µg/100 mg), and Sample B (464.02 µg/100 mg). Notably, Is 3,7 diglucoside turned out to be the most abundant flavonoid in each cultivar investigated (683.62 µg/100 mg DW in Sample A, 433.65 µg/100 mg DW in Sample B, and 644.43 µg/100 mg DW in Sample C), followed by Km 3-feruloylsophoroside-7-glucoside (72.18 µg/100 mg DW) in Sample A, Qn 3-diglucoside (31.12 µg/100 mg DW) in Sample B, and Is glycoside (49.87 µg/100 mg DW) in Sample C.

### 2.2. Determination of the Volatile Content of B. juncea Accessions Using GC-FID/MS

**Figure 3:**
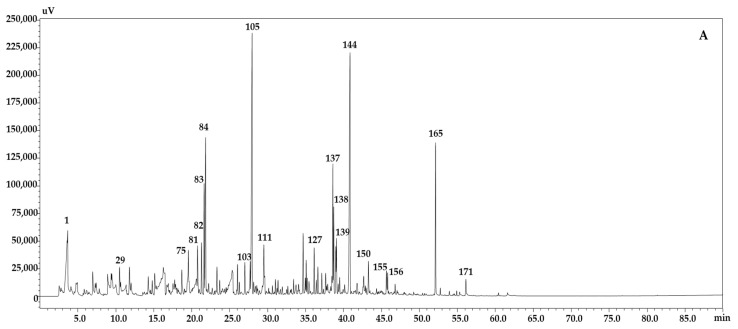

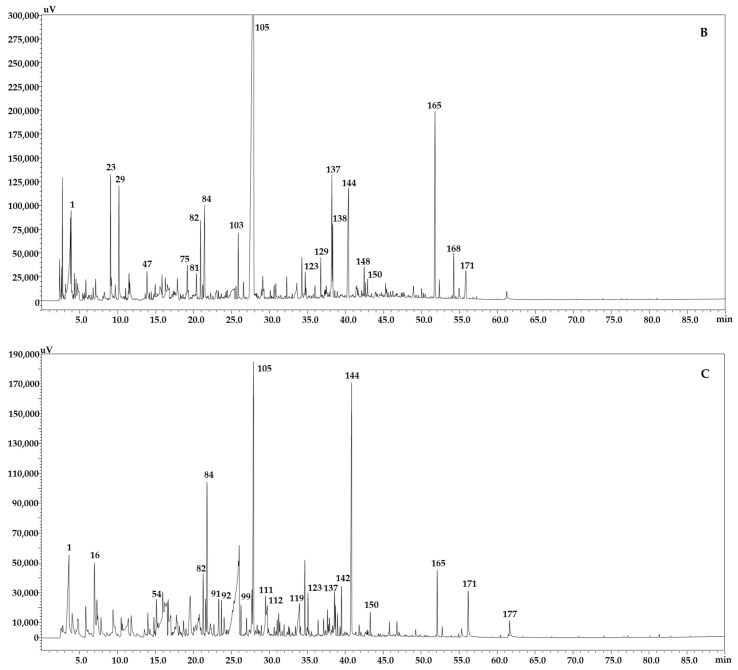
GC-MS analysis of the chemical components from extracts of the leaves of the three *Brassica* cultivars investigated, collected at the edible salad phase. (**A**) *B. juncea* ISCI 99; (**B**) *B. juncea* ISCI Top; (**C**) *B. juncea* ISCI “Broad-leaf”.

Recently, there has been more interest in the determination of organic compounds from plants and plant material, in order to evaluate their potential biological activity [29]. GC–MS is the most ideal technique for qualitative analysis of volatile and semi volatile bioactive compounds [33]. As a general rule, when using an MS detector, operating in scan mode, % abundance (% area) should not be employed, since the linear dynamic range of this detector is narrow. On the other hand, quantification is possible in Selected Ion Monitoring (SIM) mode, with selected ions calibrated with external standards. In this work, all samples were analyzed by GC-MS for compound identification, and FID for reliable peak quantification [34,35]. Figure 3 reports the GC-MS chromatograms of the volatile content of leaves of the three *Brassica* cultivars investigated, collected at the edible salad phase, when plants are about 15–20 cm high, while Table 3 reports the correspondent quantitative determination by GC-FID. It can be appreciated how all of the extracts from the three *B. juncea* accessions are characterized by mixtures of different types of organic compounds. The identified compounds include alcohols, aldehydes, esters, fatty acids, ketones, sulfur compounds, and other compounds. The major volatile constituents of *B. juncea* L. leaf extracts in the three cultivars were benzenepropanenitrile (34.94% in Sample B, 8.16% in Sample A, 6.24% in Sample C), followed by benzofuranone (8.54% in Sample A, 6.32% in Sample C, 3.64% in Sample B), and phytone (3.77% in Sample B, 2.85% in Sample A, 1.01% in Sample C) [29,36]. It is worth mentioning that the concentrations and profiles of different compounds in *Brassica* genus vary according to cultivar vegetable parts and the development stage of the plant [37]. Among alcohols, in all cultivars, the most abundant one was represented by phenethyl alcohol (4.16% in Sample A, 2.68% in Sample C, 2.39% in Sample B). Methyl benzoate (0.42% in Sample B, 0.27% in Sample C, 0.22% in Sample A) was the ester with the highest content, whereas the most abundant aldehydes, responsible for characteristic aromas [38], were represented by *n*-Nonanal in Sample B (1.24%) and Sample A (1.13%), and safranal in Sample C (1.80%). Among fatty acids (Z,Z,Z)-9,12,15-Octadecatrienoic acid was the one with major content and was detected only in Sample C (0.51%) and Sample A (0.11%). 

Interestingly, selected sulfur compounds (isothiocyanates, ITC) were also detected as they belong to major secondary metabolites of the Brassicaceae family. Among them, 2-propenyl-Isothiocyanate was the major ITC derived from aliphatic glucosinolates, and was detected in all cultivars (2.07% in Sample B, 0.74% in Sample A, 0.61% in Sample C). The content of the ITCs may vary, depending on the plant species studied, side-chain substitutions, cellular pH, and iron concentration [39,40].

### 2.3. Chemical Characterization of B. juncea DSMs

The chemical characterization of *B. juncea* DSMs is summarized in Table 4. Proteins were the main component of *B. juncea* DSMs; the results for Sample A are very similar to those of plants belonging to the Fabaceae family, such as soy [41]. The glucosinolate (GSL) analysis accounted for a maximum total of 205.4 µmol/g in Sample A and revealed a very similar profile in GSLs. Sample A and Sample B are characterized in particular by 97% of 2-propenyl GSL, 5% of but-3-enyl GSL, and only 2–3% of 4-hydroxy-3-indolylmethyl GSL. The Sample C selection is characterized by a higher percentage (%) in but-3-enyl GSL, in comparison to the other two cultivars.

## 3. Materials and Methods

### 3.1. Chemical and Reagents

LC-MS grade water, methanol, acetonitrile, and acetic acid were obtained from Merck Life Science (Merck KGaA, Darmstadt, Germany). Km 3-*O*-glucoside, Is 3-*O*-glucoside and Qn 3-*O*-glucoside were obtained from Merck Life Science (Merck KGaA, Darmstadt, Germany). Stock solutions of 1000 mg/L were prepared for each standard by dissolving 10 mg in 10 mL of methanol.

### 3.2. Plant Material

*B. juncea* selections were provided by the *Brassica* seed collection of CREA-CI [42]. They were sown in autumn on 15 October 2017, each in a 30 m^2^ plot, at the CREA experimental farm located at Budrio (Bologna) in the Po Valley area (Emilia Romagna region, 44°32′00″ N; 11°29′33″ E, altitude 28 m a.s.l.). The area was characterized by flat land with alluvial deep loamy soil, with a medium level of total nitrogen content and organic matter content. The cultivation was carried out without fertilization, and it did not require other agronomical input until harvest. Plant samples were collected at three different phenological phases: (i) the first phase, 12 ± 3 cm (Sample A) to 23 ± 3 cm (Sample B and Sample C) high, the edible salad phase; (ii) the second phase, 18 ± 2 cm (Sample A) to 33 ± 4 cm (Sample B and Sample C) high, the culmination edible salad phase, when stems and leaves started to have the same weight; and (iii) the third phase, when inflorescence was completely developed. For each sampling time, six different plants (randomly chosen) were manually harvested, brushed (to physically remove soil residue), and collected, distinguishing the different tissues (leaves, stems, roots, and flowers). Samples were immediately frozen and freeze-dried for storage in glass vacuum desiccators. Lyophilized tissues were finely powdered to 0.5 µm size for analysis.

### 3.3. Seed Cake Preparation and Main Characterization

Seed cake from *B. juncea* is the major by-product from this oilseed crop, and, to date, oil yield is its main economic value [43]. Seeds were extracted overnight at room temperature with n-hexane (1:10 *w*/*v*), in a rotary shaker. The aim was to preserve the largest number of bioactive molecules. Seed cake was pested ground in a mortar and left to dry at 40 °C until constant weight, and finally it was ground to 0.5 mm size. The *B. juncea* defatted seed meals (DSMs) were characterized for moisture, nitrogen, residual oil, and glucosinolate (GSL) content. Moisture content was determined by evaluating the difference between its weight before and after oven drying at 105 °C for 12 h. Total nitrogen content was determined according to the American Society for Testing Materials (ASTM D5373 2016) [44], and the crude protein content was expressed as a percentage of dry matter and calculated from nitrogen using the conventional factor for soy proteins of 6.25 [45]. Residual oil was extracted by a standard automated continuous extraction, following the Twisselmann principle, by using an E-816 Economic Continuous Extraction (ECE) unit (BÜCHI Labortechnik AG, Flawil, Switzerland), and hexane as solvent. GSL content and profiles were determined by HPLC-UV analysis of desulfo-GSLs following the ISO 9167-1 method (ISO 9167-1:1992/Amd 1:2013) [46]. The desulfo-GSLs were detected monitoring their absorbance at λ = 229 nm and identified with respect to their UV spectra and retention times [47,48]; their amounts were estimated using sinigrin as the external standard. Each extraction and analysis was performed in triplicate.

### 3.4. Sample Preparation

#### 3.4.1. HPLC-PDA-MS

**Figure 4:**
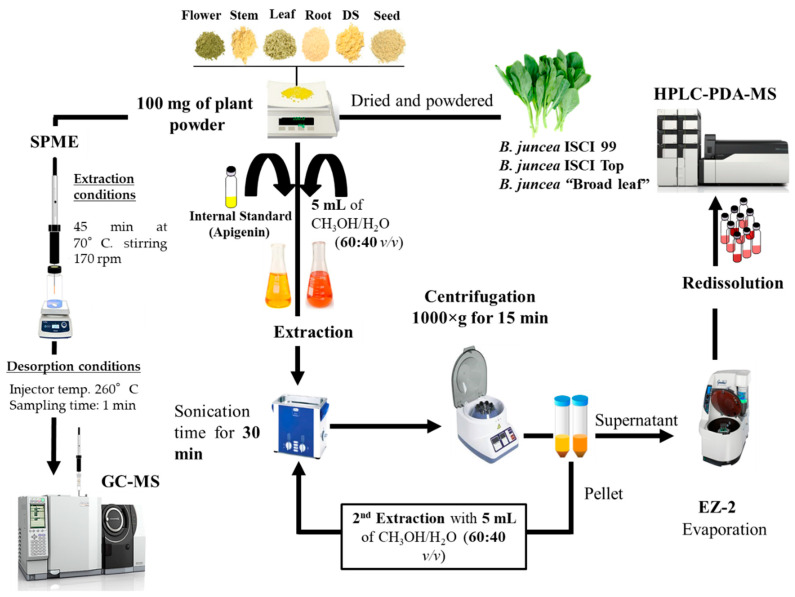
Extraction of the metabolite content of *B. juncea* cultivars prior to HPLC-PDA-MS/MS analyses.

Extraction of the metabolite content was carried out based on the following protocol [7], with some modifications. All samples were spiked prior the extraction with 50 µL of apigenin (1000 ppm), which was evaporated with the use of nitrogen. The powder of different plant parts (seed, root, stem, leaf, and flower) of *B. juncea,* besides the DSM of the three different cultivars, were weighed into 100 mg. The samples were extracted twice with 5 mL of a mixture of methanol:water (60:40, *v/v*) for 30 min in a sonicator and centrifuged at 1000× *g* for 15 min, followed by filtration of the supernatants through 0.45 µm filter paper; Merck Life Science (Merck KGaA, Darmstadt, Germany). The prepared organic extracts were subjected to evaporation in an EZ-2 evaporator, and then redissolved in 1 mL of the same solvent mixture of extraction methanol:water (60:40 *v/v*). A total of 10 µL was injected. The whole process is illustrated in Figure 4.

#### 3.4.2. GC-FID/MS

Extraction of the volatile compounds was performed with mean of a DVB/CAR/PDMS (SPME fiber) of 50/30 µm (Merck Life Science, Merck KGaA, Darmstadt, Germany). The conditioning of the SPME was carried out according to Merck Life Science’s recommendations, through its insertion into the GC injector at 270 °C for 30 min. A total of 250 mg of each *B. juncea* sample was placed into a 20 mL sealed vial with a magnetic cap, with silicone/PTFE septa (Agilent Technologies, Santa Clara, CA, USA). The sample was stirred at 170 rpm at a temperature of 70 °C for 45 min. The SPME fiber was exposed to the GC injector at a temperature of 260 °C for 1 min, following by the exposition of the fibers to the headspace for 45 min, in the same above-mentioned conditions. The extracted volatile compounds that occurred in the fiber were introduced to the GC injector for thermal desorption.

### 3.5. Instrumentation

#### 3.5.1. HPLC-PDA-MS

Analyses were performed on a Shimadzu system (Kyoto, Japan), consisting of a CBM-20A controller, two LC-30AD dual-plunger parallel-flow pumps, a DGU-20A_5_R degasser, a CTO-20AC column oven, a SIL-30AC autosampler, and an SPD-M30A PDA detector (1.0 μL detector flow cell volume). The LC system was hyphenated to an LCMS-8050 triple quadrupole mass spectrometer through an ESI source (Shimadzu, Kyoto, Japan). For data handling, the Shimadzu LabSolutions software (version 5.93) (Kyoto, Japan) was employed.

#### 3.5.2. GC-FID/MS

Compound identification was carried out on a GCMS-QP2010 system (Shimadzu, Kyoto, Japan) equipped with a split–splitless injector. Data files were collected and elaborated by using Shimadzu “GCMS solution” software (version 4.45) (Kyoto, Japan).

Compound quantification was performed on a GC2010 system (Shimadzu, Kyoto, Japan) equipped with a split–splitless injector. Data files were collected and elaborated by using Shimadzu LabSolutions software (version 5.92) (Kyoto, Japan).

### 3.6. Analytical Conditions

#### 3.6.1. LC-PDA-MS

Analyses were performed on an Ascentis Express RP C18 column (150 × 4.6 mm, 2.7 µm I.D., Merck Life Science, Merck KGaA, Darmstadt, Germany).

The mobile phase consisted of water/acetic acid (99.85/0.15 *v/v*, solvent A) and acetonitrile (solvent B), with the following gradient elution: 0–5 min, 5% B, 5–15 min, 10% B, 15–30 min, 20% B, 30–60 min, 50% B, 60 min, 100% B.

Photodiode array detector was applied in the range of λ = 200–450 nm, where *B. juncea* polyphenols were detected at λ = 330 nm (sampling frequency: 12.5 Hz, time constant: 0.16 s). The entire LC flow was 1 mL/min and injection volume was 10 µL.

MS analysis was performed in negative and positive mode and scan range was set at *m/z* 100–1400; scan speed of 2727 amu/s. The conditions of ESI were as follows: event time 0.5 s; nebulizing gas (N_2_) flow rate 3 L/min; drying gas (N_2_) flow rate, 10 L/min; interface temperature: 300 °C; heat block temperature: 400 °C; DL (desolvation line) temperature: 250 °C; DL voltage: 1 V; interface voltage: −3 kV; Qarray DL voltage 0 V, Q3 pre-rod bias 15 V.

##### Construction of Calibration Curves

Due to the lack of commercial standards of native polyphenols, three standards, representative of the chemical classes under study were selected: Km 3-*O*-glucoside, Is 3-*O*-glucoside and Qn 3-*O*-glucoside. Standard calibration curves were prepared in a concentration range 0.1–100 mg/L with five different concentration levels. Triplicate injections were made for each level, and a linear regression was generated. The calibration curves with the external standards were obtained using concentration (mg/L) with respect to the area obtained from the integration of the PDA peaks at a wavelength of 330 nm. The amount of the compound was finally expressed in µg/100 mg DW.

#### 3.6.2. GC-FID/MS

Volatile compounds were analyzed on a GC-MS system using an SLB-5ms fused-silica capillary column (30 m × 0.25 mm i.d. × 0.25 μm df film thickness) (Merck Life Science, Merck KGaA, Darmstadt, Germany). The injection port was operated in splitless mode, at the temperature of 260 °C. Helium was kept at the linear velocity of 30.0 cm/s corresponding to an inlet pressure of 24.2 KPa. The oven temperature program was set at 40 °C (held for 1 min); it was ramped up to 350 °C (held for 5 min) at a rate of 3 °C/min. The electron impact (EI) source temperature was maintained at 220 °C and the interface was set at the temperature of 250 °C. Mass range acquisition was made in full scan mode in the mass range of 40–660 *m/z*, with an event time of 0.2 s. Compounds were identified with the support of “FFNSC 4.0” (Shimadzu Europa GmbH, Duisburg, Germany), which consisted of a library of volatile compounds obtained and stored by GC-MS separation and “W11N17” (Wiley11-Nist17, Wiley, Hoboken, NJ, USA; Mass Finder 3). Identification was performed applying a spectral similarity filter (match over 85%) using also linear retention indices (LRI) that were calculated using a C7–C30 saturated n-alkane homologue series (1000 g/mL, 49451-U) supplied by Merck Life Science, Merck KGaA, Darmstadt, Germany.

The quantification of the volatile compounds was performed on a GC-FID system using the same capillary column and temperature program employed in the qualitative analysis. The carrier gas (helium) was kept at the linear velocity of 30.0 cm/sec corresponding to an inlet pressure of 97.4 KPa and the split mode of the injector was set to splitless. The flame temperature was set at 350 °C (sampling rate 200 ms). Makeup flow was 30 mL/min and hydrogen and airflow was 40 mL/min and 400 mL/min, respectively.

## 4. Conclusions

A comprehensive characterization of the chemical profile of different tissues of *B. juncea* cultivars was reported. Specifically, leaves, stems, roots, and flowers of *B. juncea* were analyzed by HPLC-PDA/ESI-MS/MS. Moreover, the leaf extracts, which turned out to be the most complex ones in terms of volatile compounds, were analyzed by GC-FID/MS, along with a chemical characterization of defatted seed meals (DSM). As far as the volatile content was concerned, more than 179 chemical constituents were identified; on the other hand, for the non-volatile part, a total of 35 metabolites were positively identified, revealing a large number of highly glycosylated and acylated isorhamnetin, quercetin, and kaempferol derivates. Among DSMs, interestingly, proteins were the main components accounting for 44.0% DW, 37.4% DW, and 36.8% DW for Sample A, Sample B, and Sample C, respectively. Based on the phytocomponents identified, this crop could have an important application in pharmaceutical and nutraceutical fields. At the same time, differences between varieties and plant tissues demonstrate the importance of cultivar selection and validation. To this regard, further studies are necessary to evaluate the bioactivity and toxicity profile through in vitro and in vivo models of materials from the most promising varieties.

## Figures and Tables

**Table 1:** Metabolite determination of the flowers extracts of the three *B. juncea* accessions using HPLC-PDA-ESI-MS/MS.

No	Tentative ID	λ max (nm)	t_R_ (min)	[M−H]^-^	MS^2^ ions	(µg/100 mg DW)		
						Sample A	Sample B	Sample C
1	Malic Acid	215; 260	1.34	133.0	-	*	*	*
2	Citric acid	215; 260	1.53	191.0	-	*	*	*
3	Km 3-diglucoside-7-glucoside	265; 345	4.20	771.2	609	*	*	*
4	Feruloylglucose	236; 285	13.40	355.2	193	Nd	Nd	Nd
5	Qn 3-sophoroside-7-glucoside	257; 352	13.90	787.2	625	9.73 ± 0.31	2.85 ± 0.64	3.63 ± 0.07
6	Rhamnosyl-ellagic acid	283; 313	14.28	447.0	-	Nd	Nd	Nd
7	Rhamnosyl-ellagic acid	283; 313	14.55	447.0	-	*	*	*
8	Qn 3-hydroxyferuloylsophoroside-7-glucoside	247; 335	15.07	979.2	625	65.60 ± 0.50	11.17 ± 2.12	6.60 ± 0.92
9	Km 3-sophoroside-7-glucoside	266; 343	15.33	771.2	609	5.25 ± 0.06	6.60 ± 0.86	19.05 ± 0.55
10	Qn 3-caffeoylsophoroside-7-glucoside	242; 330	15.77	949.2	625	22.04 ± 0.75	9.69 ± 2.32	5.53 ± 0.17
11	Km 3-hydroxyferuloylsophoroside-7-glucoside	232; 330	16.65	963.2	609	35.52 ± 0.07	8.16 ± 2.22	6.58 ± 0.00
12	Feruloylglucose	236; 326	16.88	355.2	193	*	*	*
13	Km 3-caffeoyldiglucoside-7-glucoside	233; 330	17.57	933.2	-	5.20 ± 0.10	4.91 ± 1.21	8.57 ± 0.04
14	Qn 3-diglucoside	256; 360	17.76	625.1	463; 301	71.75 ± 3.11	31.12 ± 8.80	40.05 ± 0.60
15	Qn 3-sinapoyltriglucoside-7-glucoside	238; 330	17.90	1155.3	993	Nd	Nd	21.86 ± 0.34
16	Qn 3-sinapoyltriglucoside-7-glucoside	245; 340	18.03	1155.3	993	49.06 ± 0.89	18.83 ± 3.89	Nd
17	Km 3-hydroxyferuloylsophoroside-7-glucoside	254; 338	18.51	963.2	625	35.62 ± 0.08	9.87 ± 2.66	5.93 ± 0.06
18	Km 3-hydroxyferuloylsophorotrioside-7-glucoside	268; 330	18.53	1125.3	963	Nd	Nd	Nd
19	Km 3-sinapoylsophorotrioside-7-glucoside	268; 330	18.78	1139.3	771	Nd	14.12 ± 3.33	23.17 ± 0.55
20	Km 3-sinapoylsophorotrioside-7-glucoside	268; 330	19.29	1139.3	771	1.80 ± 0.06	7.85 ± 2.02	10.72 ± 0.11
21	Km 3-sinapoylsophoroside-7-glucoside	268; 333	19.50	977.2	609; 815	18.69 ± 0.10	5.36 ± 1.30	5.29 ± 0.00
22	Km 3-feruloylsophoroside-7-glucoside	266; 341	20.26	947.2	609	72.18 ± 0.08	29.23 ± 8.00	25.17 ± 0.50
23	Is 3,7-diglucoside	252; 352	21.20	639.1	477; 315	683.62 ± 1.14	433.65 ± 2.94	644.43 ± 0.63
24	Feruloyl malate	242; 323	24.23	309.1	-	*	*	*
25	Sinapic acid	270; 326	24.27	223.1	179	Nd	Nd	Nd
26	Sinapoyl malic acid	240; 326	25.05	339.1	223	*	*	*
27	Sinapoyl-feruloyl-triglucoside	280; 325	25.21	885.3	499	Nd	Nd	Nd
28	Sinapoyl-hydroxyferuloyl-diglucoside	244; 330	29.46	739.2	515	*	*	*
29	Isorhamnetin glucoside	256; 351	31.94	477.1	-	48.63 ± 0.10	30.37 ± 8.49	49.87 ± 0.07
30	Km glucoside	269; 330	31.49	447.0	-	Nd	Nd	Nd
31	Disapoyl-gentiobiose	240; 330	33.32	753.2	529; 499	*	*	*
32	Sinapoyl-feruloyl-gentiobiose	240; 330	34.20	723.2	529; 499	*	*	*
33	Diferuloyldiglucoside	240; 326	34.82	693.1	499	*	*	*
34	Trisinapoylgentiobiose	240; 326	36.53	959.3	735; 529	*	*	*
35	Feruoyl-disapoyl-gentiobiose	240; 326	37.28	929.3	705; 511	*	*	*

Nd: not detected. * Not quantified in absence of standard. DW: Dry weight.

**Table 2:** Quantitative performance of the flavonoidic reference materials used in this study.

Reference Material	Standard Curve	R^2^	LoD (μg/mL)	LoQ (μg/mL)	Precision (RSD, %)
Qn 3-*O*-glucoside	y = 13,424x + 898.59	0.9939	0.013	0.043	0.41
Is 3-*O*-glucoside	y = 14,948x − 2966.9	0.9963	0.048	0.159	0.34
Km 3-*O*-glucoside	y = 17,660x – 10,681	0.9963	0.021	0.072	0.36

**Table 3:** Quantitative determination by GC-FID of the chemical components of the leaves of the three Brassica accessions investigated. FFNSC: Flavor and Fragrance Natural and Synthetic Compounds; LRI: Linear Retention Indices.

							Sample A	Sample B	Sample C
N.	Compounds Name	Lib. Name	Id. Method	Similarity	LRI Lib	LRI Exp	Area %	Area %	Area %
1	Ethanoic acid	FFNSC 4.0	MS, LRI	98	661	665	4.83	4.18	4.66
2	2-Butenenitrile	W11N17	MS, LRI	90	664	675	0.27		0.44
3	Hydroxyacetone	FFNSC 4.0	MS, LRI	94	682	684	0.07	Nd	0.15
4	3-hydroxy-Pentene	FFNSC 4.0	MS, LRI	92	691	691	0.06	Nd	0.20
5	3-Pentenone	FFNSC 4.0	MS, LRI	93	677	693	Nd	0.45	Nd
6	*n*-Pentanal	FFNSC 4.0	MS, LRI	94	696	701	0.52	0.41	0.40
7	methyl-Thiocyanate	FFNSC 4.0	MS, LRI	91	710	711	Nd	0.54	Nd
8	Propionic acid	FFNSC 4.0	MS, LRI	91	698	711	0.53	0.21	0.38
9	Acetoin	FFNSC 4.0	MS, LRI	90	716	716	Nd	Nd	0.05
10	Methyl propenyl ketone	FFNSC 4.0	MS, LRI	91	733	737	0.01	0.13	0.01
11	dimethyl-Disulfide	FFNSC 4.0	MS, LRI	93	722	741	0.08	Nd	0.01
12	(E)-2-Pentenal	FFNSC 4.0	MS, LRI	92	751	753	0.17	0.31	0.17
13	Isobutyric acid	FFNSC 4.0	MS, LRI	91	774	758	0.07	0.06	Nd
14	Senecionitrile	FFNSC 4.0	MS, LRI	93	756	760	Nd	Nd	0.05
15	Pentyl alcohol	FFNSC 4.0	MS, LRI	96	763	767	0.13	0.07	0.05
16	2,3-Butadienol	FFNSC 4.0	MS, LRI	96	788	788	0.58	0.20	1.80
17	*n*-Hexanal	FFNSC 4.0	MS, LRI	98	801	802	0.36	0.37	0.34
18	3-Butenoic acid	W11N17	MS	91	-	806	Nd	0.30	Nd
19	Butyric acid	FFNSC 4.0	MS, LRI	93	818	808	0.10	Nd	Nd
20	2-methyl-Pyrazine	FFNSC 4.0	MS, LRI	95	820	828	0.07	0.07	Nd
21	Furfural	FFNSC 4.0	MS, LRI	92	845	831	0.10	0.29	0.15
22	Sclerosol	FFNSC 4.0	MS, LRI	96	827	841	0.50	0.04	0.15
23	(E)-2-Hexenal	FFNSC 4.0	MS, LRI	95	850	852	0.50	1.76	0.57
24	(Z)-3-Hexenol	FFNSC 4.0	MS, LRI	95	853	854	0.61	0.51	0.33
25	Isovaleric acid	FFNSC 4.0	MS, LRI	94	842	860	1.02	0.07	0.17
26	2-methyl-Butyric acid	FFNSC 4.0	MS, LRI	89	881	867	0.73	0.21	0.29
27	allyl-Thiocyanate	FFNSC 4.0	MS, LRI	94	865	869	0.17	0.24	0.10
28	*n*-Hexanol	FFNSC 4.0	MS, LRI	91	867	869	0.26	0.20	0.03
29	2-propenyl-Isothiocyanate	FFNSC 4.0	MS, LRI	96	876	880	0.74	2.07	0.61
30	1-(3-methylenecyclopentyl)-Ethanone	W11N17	MS	91	-	884	0.47	0.23	0.34
31	*n*-Heptanal	FFNSC 4.0	MS, LRI	97	906	902	Nd	0.17	0.16
32	*n*-Pentanoic acid	FFNSC 4.0	MS, LRI	92	911	903	0.90	0.20	1.27
33	3-methyl-Crotonic acid	FFNSC 4.0	MS, LRI	91	907	905	0.12	Nd	0.02
34	2-butoxy-Ethanol	W11N17	MS, LRI	95	906	907	0.08	0.06	Nd
35	2-acetyl-Furan	FFNSC 4.0	MS, LRI	94	913	910	0.10	0.14	Nd
36	2(5H)-Furanone	FFNSC 4.0	MS, LRI	91	907	911	Nd	Nd	0.03
37	γ-Butyrolactone	FFNSC 4.0	MS, LRI	95	910	912	0.89	0.45	0.30
38	2,5-dimethyl-Pyrazine	FFNSC 4.0	MS, LRI	92	912	916	0.33	0.41	0.09
39	1,1′-sulfonylbis-Methane	W11N17	MS, LRI	95	922	916	0.15	0.10	Nd
40	methyl-Hexanoate	FFNSC 4.0	MS, LRI	92	922	924	0.07	0.03	0.02
41	*sec*-butyl-Isothiocyanate	FFNSC 4.0	MS, LRI	96	929	929	0.10	0.03	0.05
42	2,7-dimethyl-Oxepine	W11N17	MS, LRI	88	944	931	0.03	0.02	0.01
43	1-butoxy-2-Propanol	W11N17	MS, LRI	91	945	938	0.02	0.01	Nd
44	dihydro-3-methyl-2(3H)-Furanone	W11N17	MS, LRI	94	941	948	0.05	0.02	Nd
45	γ-Pentalactone	FFNSC 4.0	MS, LRI	91	954	953	0.16	0.02	0.06
46	(E)-2-Heptenal	FFNSC 4.0	MS, LRI	95	956	956	0.05	0.11	0.41
47	Benzaldehyde	FFNSC 4.0	MS, LRI	98	960	963	0.54	0.66	0.23
48	*N*-2-propenyl-Acetamide	W11N17	MS	94	-	964	0.11	Nd	0.07
49	Dimethyl trisulfide	FFNSC 4.0	MS, LRI	97	969	970	0.12	0.12	0.04
50	*n*-Heptanol	FFNSC 4.0	MS, LRI	92	970	970	0.04	0.02	0.02
51	3,5,5-trimethyl-2-Hexene	W11N17	MS, LRI	92	985	974	0.33	0.14	0.38
52	1-Octen-3-one	FFNSC 4.0	MS, LRI	91	973	977	0.03	0.03	0.12
53	Vinyl amyl carbinol	FFNSC 4.0	MS, LRI	94	978	979	Nd	0.10	Nd
54	3-butenyl-Isothiocyanate	FFNSC 4.0	MS, LRI	91	978	980	Nd	Nd	0.70
55	6-methyl-Hept-5-en-2-one	FFNSC 4.0	MS, LRI	95	986	985	0.32	0.29	0.31
56	2-pentyl-Furan	FFNSC 4.0	MS, LRI	92	991	989	0.34	0.15	Nd
57	2,3,5-trimethyl-Pyrazine	FFNSC 4.0	MS, LRI	90	1002	1001	1.08	0.30	Nd
58	2-ethyl-,5-methyl-Pyrazine	FFNSC 4.0	MS, LRI	88	1005	1001	Nd	0.19	Nd
59	2-ethyl-, 6-methyl-Pyrazine	FFNSC 4.0	MS, LRI	91	1000	1002	0.47	Nd	Nd
60	*n*-Octanal	FFNSC 4.0	MS, LRI	91	1006	1004	0.45	0.14	1.06
61	(E,E)-2,4-Heptadienal	FFNSC 4.0	MS, LRI	95	1013	1011	0.04	0.59	0.82
62	*n*-Hexanoic acid	FFNSC 4.0	MS, LRI	97	997	1013	2.45	0.64	3.09
63	2-ethenyl-6-methyl-pyrazine	W11N17	MS, LRI	82	1031	1020	0.36	0.54	Nd
64	(Z)-3-Hexenoic acid	FFNSC 4.0	MS, LRI	94	996	1022	0.47	0.74	1.07
65	2-ethyl-Hexanol	FFNSC 4.0	MS, LRI	88	1030	1030	0.33	0.20	Nd
66	Benzyl alcohol	FFNSC 4.0	MS, LRI	90	1040	1037	0.41	0.22	0.17
67	(E)-2-Hexenoic acid	FFNSC 4.0	MS, LRI	90	1036	1039	0.34	0.32	0.05
68	Oct-3-en-2-one	FFNSC 4.0	MS, LRI	93	1036	1040	0.22	0.18	0.11
69	Phenylacetaldehyde	FFNSC 4.0	MS, LRI	97	1045	1043	Nd	0.51	0.12
70	γ-Hexalactone	FFNSC 4.0	MS, LRI	98	1060	1054	0.68	0.11	0.39
71	(E)-2-Octenal	FFNSC 4.0	MS, LRI	90	1058	1061	0.22	0.11	0.20
72	α-Phenylethanol	FFNSC 4.0	MS, LRI	94	1064	1063	0.10	0.05	0.07
73	Acetophenone	FFNSC 4.0	MS, LRI	91	1068	1065	Nd	0.12	0.12
74	2-acetyl-Pyrrole	FFNSC 4.0	MS, LRI	94	1060	1068	0.30	0.15	0.46
75	(E,E)-3,5-Octadien-2-one	W11N17	MS, LRI	90	1073	1071	1.07	0.53	1.16
76	*n*-Octanol	FFNSC 4.0	MS, LRI	92	1076	1073	0.35	0.19	0.44
77	*p*-Cresol	FFNSC 4.0	MS, LRI	89	1072	1077	Nd	Nd	0.12
78	2-Pyrrolidone	FFNSC 4.0	MS, LRI	92	1070	1078	0.46	0.02	Nd
79	2-ethyl-, 3,6-dimethyl-pyrazine	FFNSC 4.0	MS, LRI	87	1079	1080	0.15	0.05	Nd
80	*n*-Heptanoic acid	FFNSC 4.0	MS, LRI	88	1116	1092	0.90	0.28	1.06
81	3,5-Octadien-2-one	W11N17	MS, LRI	90	1091	1095	1.15	0.56	0.60
82	*n*-Nonanal	FFNSC 4.0	MS, LRI	95	1107	1104	1.13	1.24	0.93
83	2,6-dimethyl-Cyclohexanol	W11N17	MS, LRI	89	1112	1113	2.19	0.27	0.58
84	Phenethyl alcohol	FFNSC 4.0	MS, LRI	95	1113	1117	4.16	2.39	2.68
85	methyl-Octanoate	FFNSC 4.0	MS, LRI	92	1125	1124	0.20	Nd	0.05
86	Isophorone	FFNSC 4.0	MS, LRI	88	1123	1126	0.41	0.08	0.11
87	2-Heptenoic acid	W11N17	MS	93	-	1130	Nd	Nd	0.24
88	2-nitro-Phenol	W11N17	MS, LRI	96	1135	1131	Nd	0.16	Nd
89	Benzene acetonitrile	FFNSC 4.0	MS, LRI	91	1138	1138	Nd	0.09	Nd
90	Oxophorone	FFNSC 4.0	MS, LRI	90	1148	1147	0.48	0.18	0.43
91	2,3-dihydro-3,5-dihydroxy-6-methyl-4*H*-Pyran-4-one	W11N17	MS, LRI	93	1151	1148	0.20	0.16	0.49
92	(E,Z)-2,6-Nonadienal	FFNSC 4.0	MS, LRI	93	1153	1152	Nd	0.15	0.48
93	γ-Heptalactone	FFNSC 4.0	MS, LRI	91	1155	1153	0.05	Nd	Nd
94	Menthone	FFNSC 4.0	MS, LRI	90	1158	1156	Nd	0.05	Nd
95	(E)-2-Nonenal	FFNSC 4.0	MS, LRI	90	1163	1159	Nd	0.11	0.26
96	2,2,6-trimethyl-1,4-Cyclohexanedione	W11N17	MS, LRI	89	1183	1172	0.22	Nd	Nd
97	2,4-dimethyl-Benzaldehyde	FFNSC 4.0	MS, LRI	91	1190	1175	0.42	0.16	Nd
98	Menthol	FFNSC 4.0	MS, LRI	97	1184	1178	0.47	0.22	Nd
99	*n*-Octanoic acid	FFNSC 4.0	MS, LRI	95	1192	1192	2.33	1.25	6.63
100	*n*-Dodecane	FFNSC 4.0	MS, LRI	97	1200	1200	0.10	0.13	Nd
101	Safranal	FFNSC 4.0	MS, LRI	97	1201	1203	0.61	1.01	1.15
102	*n*-Decanal	FFNSC 4.0	MS, LRI	96	1208	1207	0.29	0.07	1.80
103	β-Cyclocitral	FFNSC 4.0	MS, LRI	90	1223	1224	0.74	0.05	0.34
104	3-ethyl-4-methyl-1*H*-Pyrrole-2,5-dione	W11N17	MS, LRI	92	1239	1239	0.88	Nd	1.00
105	Benzenepropanenitrile	W11N17	MS, LRI	98	1244	1244	8.16	34.94	6.24
106	2-Phenethyl acetate	FFNSC 4.0	MS, LRI	95	1257	1257	0.20	0.10	0.24
107	β-Cyclohomocitral	FFNSC 4.0	MS, LRI	90	1256	1257	Nd	0.12	Nd
108	γ-Octalactone	FFNSC 4.0	MS, LRI	95	1263	1259	0.14	Nd	Nd
109	Benzeneacetic acid	W11N17	MS, LRI	88	1262	1259	Nd	Nd	0.09
110	2-phenyl-Crotonaldehyde	FFNSC 4.0	MS, LRI	88	1272	1273	0.29	0.22	Nd
111	3,3-dimethyl-2,7-Octanedione	W11N17	MS, LRI	88	1290	1277	1.46	0.45	0.96
112	*n*-Nonanoic acid	FFNSC 4.0	MS, LRI	96	1289	1280	0.48	0.31	1.18
113	Menthyl acetate	FFNSC 4.0	MS, LRI	92	1290	1289	Nd	0.05	Nd
114	Isobornyl acetate	FFNSC 4.0	MS, LRI	95	1287	1291	0.16	Nd	Nd
115	(E)-Cinnamonitrile	FFNSC 4.0	MS, LRI	96	1294	1295	0.14	0.15	0.09
116	*n*-Tridecane	FFNSC 4.0	MS, LRI	94	1300	1298	Nd	0.07	Nd
117	4-vinyl-Guaiacol	FFNSC 4.0	MS, LRI	92	1309	1314	0.09	0.31	0.43
118	γ-Nonalactone	FFNSC 4.0	MS, LRI	94	1362	1364	0.36	0.10	0.41
119	*n*-Decanoic acid	FFNSC 4.0	MS, LRI	94	1398	1368	0.45	0.24	2.12
120	2,6,10-trimethyl-Dodecane	W11N17	MS, LRI	91	1366	1376	0.09	0.37	Nd
121	α-Copaene	FFNSC 4.0	MS, LRI	90	1375	1381	0.12	Nd	Nd
122	1-Tetradecene	FFNSC 4.0	MS, LRI	94	1392	1390	Nd	0.08	Nd
123	n-Tetradecane	FFNSC 4.0	MS, LRI	95	1400	1400	0.42	0.42	0.70
124	Vanillin	FFNSC 4.0	MS, LRI	88	1394	1401	0.21	0.09	0.12
125	6,10-dimethyl-2-Undecanone	W11N17	MS, LRI	94	1408	1403	0.35	0.21	0.19
126	(E)-,α-Ionone	FFNSC 4.0	MS, LRI	90	1421	1422	Nd	0.13	Nd
127	(E)-Caryophyllene	FFNSC 4.0	MS, LRI	95	1424	1427	0.96	Nd	Nd
128	β-Gurjunene	FFNSC 4.0	MS, LRI	93	1437	1439	0.57	Nd.	Nd
129	(E)-Geranylacetone	FFNSC 4.0	MS, LRI	94	1450	1449	0.47	0.73	0.46
130	2,6,10-Trimethyltridecane	W11N17	MS, LRI	93	1449	1461	0.26	0.18	Nd
131	2,6-bis(1,1-dimethylethyl)-2,5-Cyclohexadiene-1,4-dione	W11N17	MS, LRI	90	1471	1461	Nd	0.17	Nd
132	2,6-Di-tert-butyl-4-hydroxy-4-methylcyclohexa-2,5-dien-1-one	W11N17	MS, LRI	91	1475	1463	0.31	Nd	0.63
133	Phenylethyl isothiocyanate	FFNSC 4.0	MS, LRI	95	1464	1470	0.32	0.25	0.79
134	1-chloro-Dodecane	W11N17	MS, LRI	92	1469	1471	Nd	0.10	Nd
135	Dodecanol	FFNSC 4.0	MS, LRI	94	1476	1477	0.23	Nd	0.36
136	4-(2,6,6-Trimethylcyclohexa-1,3-dienyl)but-3-en-2-one	W11N17	MS, LRI	93	1485	1482	0.41	0.20	0.23
137	(E)-,β-Ionone	FFNSC 4.0	MS, LRI	93	1482	1485	2.53	2.44	0.89
138	Ionone epoxide	FFNSC 4.0	MS, LRI	90	1483	1488	1.59	1.16	0.65
139	1-Pentadecene	W11N17	MS, LRI	96	1492	1493	0.91	0.19	0.56
140	β-Selinene	FFNSC 4.0	MS, LRI	95	1492	1498	1.10	Nd	Nd
141	*n*-Pentadecane	FFNSC 4.0	MS, LRI	96	1500	1500	0.26	0.15	0.35
142	Unknown	-	-	-	-	-	-	-	-
143	2,4-bis(1,1-dimethylethyl)-Phenol	W11N17	MS, LRI	92	1513	1507	Nd	0.09	Nd
144	5,6,7,7a-tetrahydro-4,4,7a-trimethyl-2(4*H*)-Benzofuranone	W11N17	MS, LRI	97	1532	1544	8.54	3.64	6.32
145	*n*-Dodecanoic acid	FFNSC 4.0	MS, LRI	92	1581	1564	Nd	Nd	0.29
146	3-methyl-Pentadecane	W11N17	MS, LRI	90	1570	1571	0.07	0.10	0.19
147	2,2,4-Trimethyl-1,3-pentanediol diisobutyrate	W11N17	MS, LRI	91	1588	1585	Nd	0.17	Nd
148	2-[[[4-(4-hydroxy-4-methylpentyl)-, 3-cyclohexen-1-yl]methylene]amino]-, methyl-Benzoate	FFNSC 4.0	MS, LRI	97	1589	1586	0.22	0.42	0.27
149	*n*-Hexadecene	FFNSC 4.0	MS, LRI	86	1593	1593	0.15	0.28	0.23
150	*n*-Hexadecane	FFNSC 4.0	MS, LRI	96	1600	1601	0.79	0.38	0.72
151	1-butylheptyl-Benzene	W11N17	MS, LRI	91	1632	1633	0.09	0.06	0.16
152	Benzophenone	FFNSC 4.0	MS, LRI	96	1627	1639	0.10	0.02	0.26
153	1-propyloctyl-Benzene	W11N17	MS, LRI	93	1643	1640	0.10	0.12	0.19
154	1,1′-oxybis-Octane	W11N17	MS, LRI	91	1659	1660	Nd	0.27	0.19
155	β-Eudesmol	FFNSC 4.0	MS, LRI	89	1656	1666	0.58	Nd	Nd
156	(Z,Z,Z)-1,8,11,14-Heptadecatetraene	W11N17	MS, LRI	93	1664	1667	0.43	0.19	0.41
157	*n*-Heptadecane	FFNSC 4.0	MS, LRI	94	1700	1700	0.10	0.08	0.32
158	Tetradecanoic acid	FFNSC 4.0	MS, LRI	93	1773	1761	Nd	Nd	0.55
159	1-ethyldecyl-Benzene	W11N17	MS, LRI	90	1766	1763	0.02	0.04	Nd
160	3-methyl-Heptadecane	W11N17	MS, LRI	92	1771	1769	Nd	0.13	0.15
161	6-Hydroxy-4,4,7a-trimethyl-5,6,7,7a-tetrahydrobenzofuran-2(4H)-one	W11N17	MS, LRI	90	1784	1778	0.02	Nd	0.18
162	1-Octadecene	FFNSC 4.0	MS, LRI	95	1793	1793	0.04	0.19	0.03
163	*n*-Octadecane	FFNSC 4.0	MS, LRI	95	1800	1800	0.04	0.09	0.04
164	Neophytadiene	FFNSC 4.0	MS, LRI	90	1836	1837	0.03	Nd	0.04
165	Phytone	FFNSC 4.0	MS, LRI	91	1841	1839	2.85	3.77	1.01
166	*n*-Nonadecane	FFNSC 4.0	MS, LRI	90	1900	1897	Nd	0.01	Nd
167	methyl-7,10,13-hexadecatrienoate	W11N17	MS	91	-	1897	0.09	Nd	0.27
168	3-Methyl-2-(3,7,11-trimethyldodecyl) furan	W11N17	MS	92	-	1913	0.05	0.72	Nd
169	methyl-Hexadecanoate	FFNSC 4.0	MS, LRI	94	1925	1926	0.09	0.05	0.06
170	(Z,Z,Z)-7,10,13-Hexadecatrienoic acid	W11N17	MS, LRI	92	1945	1938	0.11	Nd	Nd
171	*n*-Hexadecanoic acid	FFNSC 4.0	MS, LRI	91	1977	1963	0.41	0.86	1.16
172	*n*-Eicosane	FFNSC 4.0	MS, LRI	94	2000	1997	Nd	0.01	Nd
173	methyl-Linoleate	FFNSC 4.0	MS, LRI	91	2093	2094	0.02	Nd	0.01
174	*n*-Heneicosane	FFNSC 4.0	MS, LRI	90	2100	2097	Nd	0.02	Nd
175	methyl-Linolenate	FFNSC 4.0	MS, LRI	93	2098	2100	0.07	0.00	0.05
176	(Z,Z)-9,12-Octadecadienoic acid	W11N17	MS, LRI	91	2133	2135	0.01	Nd	0.10
177	(Z,Z,Z)-9,12,15-Octadecatrienoic acid	W11N17	MS, LRI	93	2139	2140	0.11	Nd	0.51
178	*n*-Tetracosane	FFNSC 4.0	MS, LRI	89	2400	2397	Nd	0.00	Nd
179	*n*-Pentacosane	FFNSC 4.0	MS, LRI	92	2500	2496	Nd	0.01	0.01
180	*n*-Heptacosane	FFNSC 4.0	MS, LRI	92	2700	2700	0.01	0.02	0.03
	**Total Identified**						**83.15**	**86.37**	**75.53**

**Table 4:** *B. juncea* defatted seed meals (DSMs): main characterization. Mean values ± SD (*n* = 3) are shown. The results are expressed as percentage (%) on dry matter basis and μmoles·g^−1^ of seed meal (dry weight).

DSM	Moisture	Oil Content	Proteins		Glucosinolates	
				but-3enyl GSL	2-propenyl GSL	4-hydroxy-3-indolylmethylGSL
	(% DW)	(% DW)	(% DW)	(µmol /g)	µmol /g)	(µmol /g)
Sample A	8.3 ± 0.1	11.1 ± 0.1	44.0 ± 0.5	2.4 ± 0.2	200 ± 3	3.0 ± 0.7
Sample B	8.8 ± 0.5	15.7 ± 0.2	37.4 ± 0.2	2.1 ± 0.1	137 ± 3	2.10 ± 0.04
Sample C	8.3 ± 0.3	16.9 ± 0.1	36.8 ± 0.2	4.9 ± 0.2	148 ± 2	2.2 ± 0.2

## Supplementary Materials

The following are available online. Table S1: Metabolite determination of the ISCI 99 cultivar extracts of B. juncea collected on different phenological stages by HPLC-PDA, Table S2: Metabolite determination of the ISCI Top cultivar extracts of B. juncea collected on different phenological stages by HPLC-PDA, Table S3: Matabolite determination of the ISCI “Broad-leaf” cultivar extracts of B. juncea collected on different phenological stages by HPLC-PDA.
