# Supplementary material for: Chemical Characterization of Three Accessions of Brassica juncea L. Extracts from Different Plant Tissues
Source: Molecules. 2020 Nov 19;25(22):5421. doi: 10.3390/molecules25225421 (PMC7699538; doi:10.3390/molecules25225421)
Supplement: Supplementary file 1 [file molecules-25-05421-s001.pdf]

Table S1. Metabolite determination of the ISCI 99 cultivar extracts of *B. juncea* collected on different phenological stages by HPLC-PDA

No	(μg/100 mg DW)											
	**Leaf**	**Stem**	**Root**	**Leaf**	**Stem**	**Root**	**Leaf-flower**	**Stem-flower**	**Root-flower**	**Flower**	**Seed**	**DF**
1	*	*	*	*	*	*	*	*	*	*	Nd	Nd
2	*	Nd	*	*	*	*	*	*	*	*	Nd	Nd
3	*	Nd	Nd	*	Nd	Nd	*	*	*	*	Nd	Nd
4	Nd	*	Nd	Nd	*	*	Nd	*	*	Nd	Nd	Nd
5	8.78	1.15	Nd	9.30	0.40	Nd	9.70	2.29	Nd	9.73	Nd	Nd
6	*	*	Nd	Nd	*	Nd	Nd	*	Nd	Nd	Nd	Nd
7	*	*	Nd	Nd	*	Nd	*	*	Nd	*	Nd	Nd
8	33.46	2.38	0.25	28.43	1.53	0.35	137.67	40.35	Nd	65.60	Nd	Nd
9	17.73	Nd	Nd	2.53	Nd	Nd	10.22	Nd	Nd	5.25	Nd	Nd
10	41.39	6.49	Nd	31.46	4.39	Nd	34.06	24.80	Nd	22.04	Nd	Nd
11	31.15	Nd	Nd	25.86	Nd	Nd	153.93	Nd	Nd	35.52	Nd	Nd
12	*	*	Nd	*	*	Nd	*	*	Nd	*	Nd	Nd
13	13.35	Nd	Nd	6.31	Nd	Nd	21.15	Nd	Nd	5.20	Nd	Nd
14	25.59	1.99	Nd	22.23	0.69	Nd	54.85	28.74	Nd	71.75	Nd	Nd
15	73.61	11.79	Nd	0.51	6.34	Nd	4.41	1.75	Nd	Nd	Nd	Nd
16	69.38	Nd	Nd	48.02	Nd	Nd	29.72	32.87	Nd	49.06	Nd	Nd
17	19.00	1.59	Nd	26.31	Nd	Nd	37.01	Nd	Nd	35.62	Nd	Nd
18	Nd	Nd	Nd	Nd	2.73	Nd	Nd	14.44	Nd	Nd	Nd	Nd
19	13.82	Nd	Nd	1.39	Nd	Nd	0.93	Nd	Nd	Nd	Nd	Nd
20	21.61	1.66	Nd	7.55	1.92	Nd	3.46	2.71	Nd	1.80	Nd	Nd
21	42.31	Nd	0.92	26.93	Nd	1.04	45.50	Nd	2.27	18.69	64.38	77.69
22	40.91	2.84	Nd	28.94	2.25	Nd	112.35	21.35	Nd	72.18	5.96	23.40
23	121.48	7.13	2.09	102.88	6.96	0.90	293.01	171.30	1.10	683.62	Nd	Nd
24	*	*	Nd	*	*	Nd	*	Nd	Nd	*	Nd	Nd
25	Nd	Nd	Nd	Nd	*	Nd	Nd	Nd	Nd	Nd	*	Nd
26	*	*	Nd	*	*	Nd	*	Nd	Nd	*	Nd	Nd
27	*	*	Nd	Nd	*	Nd	Nd	Nd	Nd	Nd	Nd	Nd
28	*	*	Nd	*	*	Nd	*	*	Nd	*	Nd	Nd
29	4.11	0.91	Nd	6.15	0.77	Nd	11.24	4.52	Nd	48.63	3.11	Nd
30	Nd	Nd	Nd	Nd	Nd	Nd	3.26	Nd	Nd	Nd	Nd	Nd

31	*	*	*	*	*	*	*	*	*	*	*	*
32	*	*	*	*	*	*	*	*	Nd	*	Nd	Nd
33	*	*	*	*	*	*	*	*	Nd	*	Nd	Nd
34	*	*	*	*	*	*	*	*	*	*	*	*
35	*	*	Nd	*	*	Nd	*	*	Nd	*	Nd	Nd

Nd: not detected * not quantified DW: dry weight DF: defatted flour

Table S2. Metabolite determination of the ISCI Top cultivar extracts of *B. juncea* collected on different phenological stages by HPLC-PDA

No	(μg/100 mg DW)											
	**Leaf**	**Stem**	**Root**	**Leaf**	**Stem**	**Root**	**Leaf-flower**	**Stem-flower**	**Root-flower**	**Flower**	**Seed**	**DF**
1	*	*	*	*	*	*	*	*	*	*	Nd	Nd
2	*	*	*	*	*	*	*	*	*	*	Nd	Nd
3	*	*	*	*	*	Nd	*	*	*	*	Nd	Nd
4	Nd	*	*	Nd	*	*	Nd	*	*	Nd	Nd	Nd
5	11.48	1.72	Nd	11.36	0.75	Nd	1.79	0.73	Nd	2.85	Nd	Nd
6	Nd	*	Nd	Nd	*	Nd	Nd	Nd	Nd	Nd	Nd	Nd
7	*	*	Nd	*	*	Nd	*	Nd	Nd	*	Nd	Nd
8	8.47	Nd	Nd	10.58	1.01	Nd	46.86	1.67	1.26	11.17	Nd	Nd
9	19.57	Nd	Nd	12.08	Nd	Nd	96.78	Nd	Nd	6.60	Nd	Nd
10	34.16	9.86	Nd	27.29	4.82	Nd	23.18	8.58	Nd	9.69	Nd	Nd
11	17.75	Nd	Nd	12.41	Nd	Nd	91.25	Nd	Nd	8.16	Nd	Nd
12	*	*	Nd	*	*	Nd	*	*	Nd	*	Nd	Nd
13	12.46	Nd	Nd	13.48	Nd	Nd	23.69	Nd	Nd	4.91	Nd	Nd
14	4.68	0.28	Nd	5.75	0.73	Nd	6.70	5.30	Nd	31.12	Nd	Nd
15	Nd	Nd	Nd	Nd	Nd	Nd	96.70	6.42	Nd	Nd	Nd	Nd
16	114.09	18.94	Nd	86.24	11.77	Nd	Nd	3.96	Nd	18.83	Nd	Nd
17	13.65	Nd	Nd	10.60	Nd	Nd	13.75	Nd	Nd	9.87	Nd	Nd
18	Nd	2.36	Nd	Nd	1.46	Nd	Nd	3.85	Nd	Nd	Nd	Nd
19	38.36	Nd	Nd	13.39	Nd	Nd	6.75	Nd	Nd	14.12	Nd	Nd
20	10.11	4.19	Nd	11.68	2.75	Nd	41.39	5.42	Nd	7.85	Nd	Nd

21	58.23	Nd	1.31	27.88	Nd	1.68	22.98	Nd	0.79	5.36	119.77	97.15
22	28.73	2.25	Nd	16.21	1.55	Nd	55.51	10.73	Nd	29.23	10.33	Nd
23	120.20	14.50	1.04	155.29	27.61	0.81	392.01	222.52	0.87	433.65	Nd	Nd
24	*	*	Nd	*	*	Nd	*	*	Nd	*	Nd	Nd
25	Nd	Nd	Nd	Nd	Nd	Nd	Nd	Nd	Nd	Nd	*	Nd
26	*	Nd	Nd	*	Nd	Nd	*	Nd	Nd	*	Nd	Nd
27	Nd	*	Nd	Nd	*	Nd	Nd	*	Nd	Nd	Nd	Nd
28	*	*	Nd	*	*	Nd	*	*	Nd	*	Nd	Nd
29	5.98	1.15	Nd	5.74	1.12	Nd	15.50	3.92	Nd	30.37	3.19	Nd
30	Nd	Nd	Nd	Nd	Nd	Nd	2.68	Nd	Nd	Nd	Nd	Nd
31	*	*	*	*	*	*	*	*	*	*	*	*
32	*	*	*	*	*	*	*	*	*	*	Nd	Nd
33	*	*	*	*	*	*	*	*	*	*	Nd	Nd
34	*	*	*	*	*	*	*	*	*	*	*	*
35	*	*	Nd	*	*	Nd	*	*	Nd	*	Nd	Nd

Nd not detected * not quantified DW dry weight DF deffated flour

Table S3. Matabolite determination of the ISCI "Broad-leaf" cultivar extracts of *B. juncea* collected on different phenological stages by HPLC-PDA

No	(µg/100 mg DW)											
	**Leaf**	**Stem**	**Root**	**Leaf**	**Stem**	**Root**	**Leaf-flower**	**Stem-flower**	**Root-flower**	**Flower**	**Seed**	**DF**
1	*	*	*	*	*	*	*	*	*	*	Nd	Nd
2	*	*	*	*	*	*	*	*	*	*	Nd	Nd
3	*	*	*	*	*	*	*	*	*	*	Nd	Nd
4	Nd	*	*	Nd	*	*	Nd	*	*	Nd	Nd	Nd
5	6.49	0.61	Nd	5.75	0.52	Nd	0.69	0.29	Nd	3.63	Nd	Nd
6	Nd	Nd	Nd	Nd	Nd	Nd	Nd	Nd	Nd	Nd	Nd	Nd
7	*	*	Nd	Nd	*	Nd	Nd	*	Nd	*	Nd	Nd
8	7.05	Nd	Nd	9.11	Nd	Nd	18.18	3.93	Nd	6.60	Nd	Nd
9	62.24	Nd	Nd	31.18	Nd	Nd	127.55	Nd	Nd	19.05	Nd	Nd
10	25.29	5.72	Nd	20.26	4.30	Nd	12.91	5.59	Nd	5.53	Nd	Nd

11	42.54	Nd	Nd	58.77	Nd	Nd	65.91	Nd	Nd	6.58	Nd	Nd
12	*	*	Nd	*	*	Nd	*	*	Nd	*	Nd	Nd
13	15.44	Nd	Nd	16.05	Nd	Nd	18.50	Nd	Nd	8.57	Nd	Nd
14	3.02	0.20	Nd	17.57	0.33	Nd	4.00	1.94	Nd	40.05	Nd	Nd
15	Nd	Nd	Nd	Nd	Nd	Nd	59.96	33.50	Nd	21.86	Nd	Nd
16	80.11	11.11	Nd	69.22	15.28	Nd	Nd	Nd	Nd	Nd	Nd	Nd
17	6.64	Nd	Nd	8.51	Nd	Nd	3.07	Nd	Nd	5.93	Nd	Nd
18	Nd	1.58	Nd	Nd	1.41	Nd	Nd	1.36	Nd	Nd	Nd	Nd
19	33.04	Nd	Nd	16.22	Nd	Nd	39.66	Nd	Nd	23.17	Nd	91.49
20	43.38	2.12	Nd	32.36	1.78	Nd	38.70	4.00	Nd	10.72	Nd	Nd
21	56.27	Nd	1.16	45.45	Nd	0.75	20.52	Nd	1.85	5.29	141.16	Nd
22	46.46	2.28	Nd	49.89	1.74	Nd	48.09	4.15	Nd	25.17	13.15	Nd
23	245.61	12.13	2.29	217.29	15.30	0.71	285.08	180.28	0.88	644.43	Nd	Nd
24	*	*	Nd	*	*	Nd	*	*	Nd	*	Nd	Nd
25	Nd	Nd	Nd	Nd	Nd	Nd	Nd	Nd	Nd	Nd	*	Nd
26	*	Nd	Nd	*	Nd	Nd	*	Nd	Nd	*	Nd	Nd
27	Nd	*	Nd	Nd	*	Nd	Nd	*	Nd	Nd	Nd	Nd
28	*	*	Nd	*	*	Nd	*	*	Nd	*	Nd	Nd
29	10.31	1.00	Nd	6.48	0.76	Nd	14.68	4.42	Nd	49.87	4.81	Nd
30	2.53	Nd	Nd	1.76	Nd	Nd	3.22	Nd	Nd	Nd	Nd	Nd
31	*	*	*	*	*	*	*	*	*	*	*	*
32	*	*	*	*	*	*	*	*	Nd	*	Nd	Nd
33	*	*	*	*	*	*	Nd	*	v	*	Nd	Nd
34	*	*	*	*	*	*	*	*	*	*	*	*
35	*	*	Nd	*	*	Nd	*	*	Nd	*	Nd	Nd

Nd not detected * not quantified DW dry weight DF deffated flour
